# Machine Learning Approaches in Diagnosis, Prognosis and Treatment Selection of Cardiac Amyloidosis

**DOI:** 10.3390/ijms24065680

**Published:** 2023-03-16

**Authors:** Alessandro Allegra, Giuseppe Mirabile, Alessandro Tonacci, Sara Genovese, Giovanni Pioggia, Sebastiano Gangemi

**Affiliations:** 1 Division of Hematology, Department of Human Pathology in Adulthood and Childhood “Gaetano Barresi”, University of Messina, 98125 Messina, Italy; 2 Clinical Physiology Institute, National Research Council of Italy (IFC-CNR), 56124 Pisa, Italy; 3 Institute for Biomedical Research and Innovation (IRIB), National Research Council of Italy (CNR), 98164 Messina, Italy; 4 Allergy and Clinical Immunology Unit, Department of Clinical and Experimental Medicine, University of Messina, 98125 Messina, Italy

**Keywords:** cardiac amyloidosis, artificial intelligence, machine learning, deep learning, ATTRwt amyloidosis, AL amyloidosis, hypertrophic cardiomyopathy, multiple myeloma, diagnosis

## Abstract

Cardiac amyloidosis is an uncommon restrictive cardiomyopathy featuring an unregulated amyloid protein deposition that impairs organic function. Early cardiac amyloidosis diagnosis is generally delayed by indistinguishable clinical findings of more frequent hypertrophic diseases. Furthermore, amyloidosis is divided into various groups, according to a generally accepted taxonomy, based on the proteins that make up the amyloid deposits; a careful differentiation between the various forms of amyloidosis is necessary to undertake an adequate therapeutic treatment. Thus, cardiac amyloidosis is thought to be underdiagnosed, which delays necessary therapeutic procedures, diminishing quality of life and impairing clinical prognosis. The diagnostic work-up for cardiac amyloidosis begins with the identification of clinical features, electrocardiographic and imaging findings suggestive or compatible with cardiac amyloidosis, and often requires the histological demonstration of amyloid deposition. One approach to overcome the difficulty of an early diagnosis is the use of automated diagnostic algorithms. Machine learning enables the automatic extraction of salient information from “raw data” without the need for pre-processing methods based on the a priori knowledge of the human operator. This review attempts to assess the various diagnostic approaches and artificial intelligence computational techniques in the detection of cardiac amyloidosis.

## 1. Introduction

### General Considerations on Amyloidosis

A rare class of diseases known as amyloidoses is brought on by the extracellular deposition of amyloid, a fibrillar material created by the organism from several precursor proteins. These proteins harm essential organs by self-assembling into highly structured aberrant conformations and depositing as minute insoluble fibers. A variety of proteins can be used to create amyloid. Some varieties are passed down from one generation to the next. Others are brought on by external factors, such as chronic kidney disease or inflammatory disease, or they develop as a result of other disorders. Among the collection of diverse conditions, which includes Creutzfeldt–Jakob, Alzheimer’s, Huntington’s, and prion diseases, there are more than 20 amyloidosis diseases [1]. The affected organs determine the type and severity of amyloidosis symptoms, as some amyloidoses are localized, whereas others are systemic. As a result, some people only experience modest symptoms, while others experience serious diseases that could be deadly [2]. The clinical overlap with other illnesses is the greatest obstacle to distinguish the various amyloidoses.

Systemic amyloidosis is divided into various groups, according to a generally accepted taxonomy, based on the proteins that make up the amyloid deposits [3]. Primary amyloidosis (AL), often referred to as amyloidosis from light chains, is characterized by changes in plasma cells that produce a significant amount of abnormal antibody proteins. The most typical locations for primary amyloid deposits include the heart, liver, spleen, tongue, kidneys, gut, arteries, skin, and nerves.

Secondary amyloidosis (AA) is due to infections, persistent inflammation, certain types of tumors. In this case, the kidney is the organ that is most severely impacted, while other organs may also sustain damage.

A group of uncommon genetic disorders known as hereditary amyloidosis are brought on by abnormalities in specific blood proteins. These modified proteins are what lead to amyloid fibrils, which have an impact on the heart, kidneys, and nervous system.

A liver-produced protein called altered transthyretin (ATTRm) is the most frequently identified cause of amyloidosis. When ATTR-CM patients experience symptomatic heart failure (HF), their functional status and quality of life gradually deteriorate, and their morbidity and mortality are substantial, with high rates of hospitalization and death [4,5,6,7].

Fibrils that build up in the various organs generate ATTR-CM, a fatal disease that progresses over time [8]. In fact, the deposition of amyloid in patients with wild-type ATTR-CM is systemic and not restricted to the heart. As a result of multiorgan involvement, clinical symptoms include carpal tunnel syndrome, lumbar stenosis, renal illness, and neuropathy of variable severity [9,10]. However, to identify patients at high risk for ATTR-CM, no validated clinical decision support (CDS) or reporting methods that take into account the various comorbidities are currently available for use in healthcare system databases or at the point of care.

Wild-type ATTR-CM is frequently misdiagnosed or detected late in the illness course despite increased awareness because patients’ HF is mistakenly assigned to more widespread causes, such as hypertension or renal disease [11,12].

AL amyloidosis is a separate type of amyloidosis, as previously mentioned. It is a rare, severe, progressive, crippling, systemic disease that is somewhat more prevalent in men and has a median age at diagnosis of roughly 65 years [13,14,15,16]. However, other less frequent forms of cardiac amyloidosis have been identified (Table 1).

More than 61% of fatalities are attributable to cardiac amyloid infiltration, which is also the main factor in determining survival in individuals with AL amyloidosis. Arrhythmias or progressive heart failure are cardiovascular events that can result in mortality; however, the exact reason is frequently unknown [17]. Atrial or ventricular arrhythmia, heart failure, embolism, stroke, and conduction delays, including severe atrioventricular block, are common cardiovascular problems.

The cardiac stage of the illness at diagnosis affects median survival [18]. In modern cohorts, the median survival is close to five years [19]. Patients with less severe cardiac involvement have a median lifespan of roughly two years compared to only four months for those with highly advanced cardiac illness [20]. The overall survival of patients has increased because of the introduction of new therapeutic drugs and regimens as well as early diagnosis [21,22,23,24]. Clonal plasma cell growth is the underlying cause of AL amyloidosis, which results in amyloidogenic immunoglobulin (Ig) light chains that aggregate to form insoluble fibrils that deposit in tissues and lead to organ failure [25,26,27,28]. More than 75% of patients with AL amyloidosis experience symptoms in the heart, making it the most frequently affected organ.

Because of its insidious onset, the rate of misdiagnosis is high, the missed diagnosis rate is still very high, and it usually progresses to a late stage by the time it is diagnosed. Instead, early diagnosis is important for a number of reasons: overall survival is poor once cardiac involvement occurs; chemotherapy followed by stem cell transplantation has significantly improved prognosis for AL amyloidosis; antibody-mediated fibril phagocytosis as well as TTR gene silencers and protein stabilizers are emerging; and recent successful trials on all-cause mortality and hospitalization are evidence of these developments. In order to improve patient outcomes with this fatal condition, awareness must grow [29].

## 2. Machine Learning and Deep Learning

It is obvious from what has been discussed so far that the differential diagnosis of the various kinds of amyloidosis is difficult. This is why the potential application of artificial intelligence (AI) methods seems intriguing. In order to generate predictions or choices without being explicitly taught to do so, machine learning (ML) algorithms construct a mathematical model from sample data, which is also referred to as “training data”. The most accurate human procedures are comparable in speed and precision to automated ML analysis [30,31,32]. 

A subclass of AI, known as deep learning (DL), uses “raw data” to automatically discover important features utilizing a hierarchy of representation levels that are not explicitly created by humans, unlike in ML. Through the use of a number of levels of representation, DL enables the automatic extraction of salient information from “raw data” without the strict need for pre-processing methods based on the a priori knowledge of the human operator. The integration of DL-based predictive analytics into clinical imaging in the medical field follows a logical evolution, with advances in cardiovascular imaging now producing high-fidelity datasets with more data than those obtained from older scanner generations [33,34]. Clinical imaging integration with DL-based algorithms has the potential to automate repetitive operations, enhance disease diagnosis and prognosis, and reveal novel biomarkers linked to certain disease processes [35,36,37].

**Figure 1:**
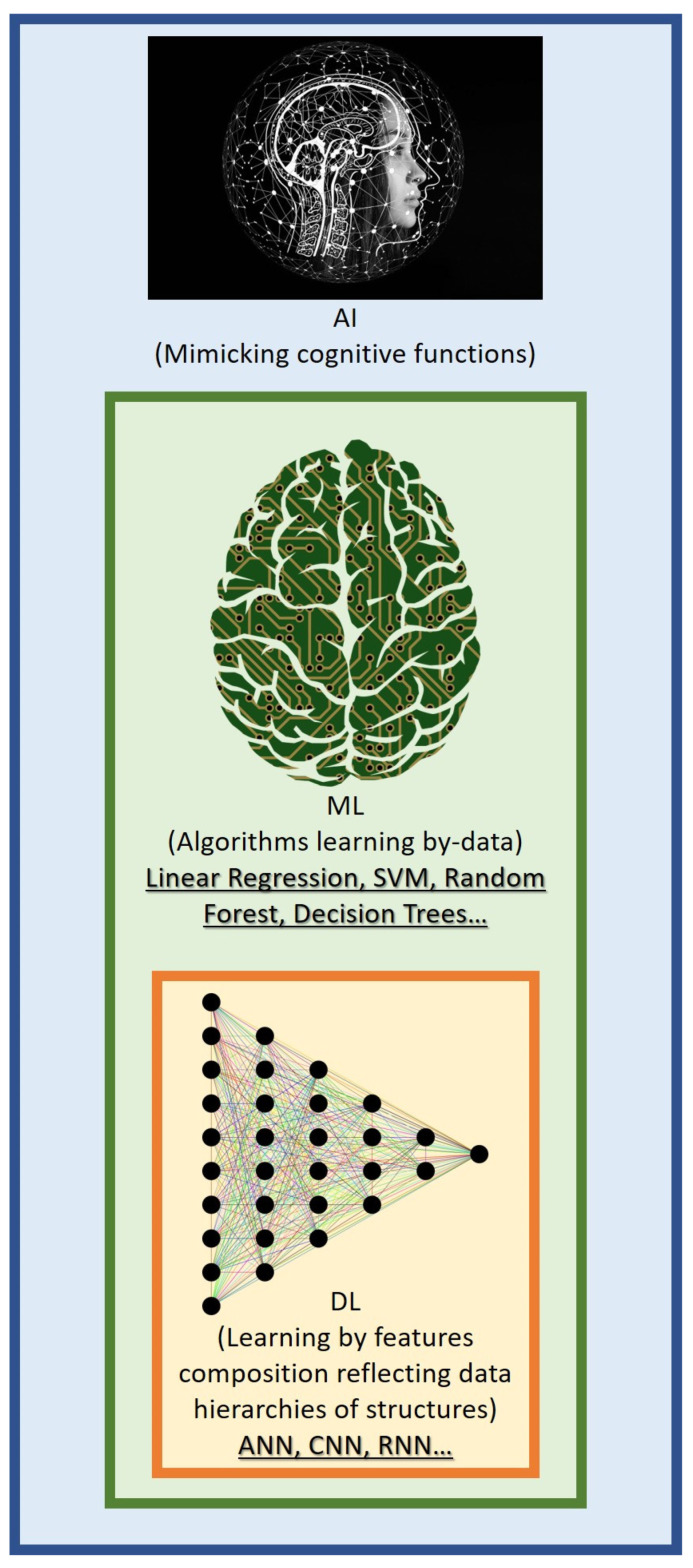
Main AI-based approaches employed in the field of amyloidosis.

The overall structure of AI-based methods employed in the field of amyloidosis, with information about the most common methods retrieved, is displayed in Figure 1.

## 3. Diagnosis of Amyloidosis

Once amyloidosis is suspected, a number of tests can be performed to confirm the diagnosis, to look for an underlying cardiopathy, and to identify the affected organs. Ig light chain abnormalities in serum can be found using free light chain (FLC) tests [38,39,40]. Immunofixation electrophoresis assays on serum and urine are used to measure and categorize aberrant Ig light chains or monoclonal proteins [41]. Positive outcomes from these assays would point to the presence of multiple myeloma, indolent B cell lymphoma, Waldenström macroglobulinemia, or AL amyloidosis [42]. In the majority of individuals with AL amyloidosis, the combination of these assays can be quite successful in detecting amyloidogenic light chains [42].

Confirmation of the diagnosis requires Congo red staining of a tissue biopsy sample. Amyloid deposits can be found quickly and safely with abdominal fat pad biopsies [43]. However, although the diagnosis of amyloidosis is confirmed by the presence of amyloid deposits by Congo red staining in fat pads, their absence does not rule out illness. A different key step in the diagnosis of AL amyloidosis is a bone marrow examination. About 85% of patients with AL amyloidosis will be detected using a combination of fat and bone marrow biopsies [44].

As for other forms of amyloidosis, in a significant, prospective, observational analysis of patients with ATTRwt-CM, the median diagnostic delay was 39 months, and 42% of patients experienced a diagnosis delay of more than 4 years [45]. Delayed ATTR-CM diagnosis can cause more advanced disease at diagnosis and result in needless consultations, testing, and treatment before diagnosis [46,47]. 

Cardiac involvement is frequent in both AL and ATTR amyloidosis, is associated with a worse prognosis, and has significant therapeutic implications [48]. CA is more likely to occur in patients with hypertrophic cardiomyopathy (HCM), and these two disorders can be misdiagnosed in a patient [49]. An estimated 1 in 500 to 1 in 5000 people in the general population have HCM. 

Diagnoses for patients older than 60 years of age grew from 9.2% in 2000 to 31.8% after 2010, and the prevalence of diagnoses for patients older than 70 years of age climbed to 10.7% after 2010. Older patients were more frequently genotype-negative and more frequently affected by sporadic manifestations of the disease [50].

Due to major improvements in diagnostic and treatment approaches over the past few years, interest in an early identification of the disease—traditionally regarded as rare and incurable—has drastically transformed [51]. The most important diagnostic advancements are in the area of ATTR-CA, whose diagnostic process has been significantly changed by the ability to obtain a non-biopsy diagnosis by cardiac scintigraphy with bone tracers. This method is the cornerstone of an algorithm that has been tested on a sizable cohort of CA patients, with results showing 99% sensitivity, 86% specificity, and a positive predictive value that is very close to 100% when used in conjunction with serum/urine immunofixation and the detection of free light chains, which rule out the presence of monoclonal proteins [52]. As a result, diagnoses of ATTR-CA have dramatically increased, and patients are now identified earlier in the course of the disease.

The identification of clinical features, electrocardiographic (ECG), and imaging findings suggestive or compatible with CA is the first step in the diagnostic process. Amyloid deposition must frequently be shown histologically, with the exception of cases where diphosphonate scintigraphy reveals a significant increase in myocardial uptake without the presence of a monoclonal gammopathy (Perugini scores 2–3) [2]. Cardiovascular magnetic resonance (CMR) has a special ability that makes it possible to characterize cardiac tissue, although its function in this diagnostic flowchart is not entirely clear [52]. A pattern of fluctuating biventricular pseudohypertrophy with widespread subendocardial-to-transmural late gadolinium enhancement (LGE) is one of several CMR findings that are quite distinctive for CA. The extent of LGE and the degree of wall thickness growth are related to the level of amyloid fiber infiltration in the myocardium. In fact, in individuals with early disease stages, amyloid deposition is restricted to the sub-endocardium of a small number of cardiac segments and becomes increasingly diffuse [53]. The myocardial and blood-pool gadolinium kinetics are entirely disrupted in the final phases of amyloid infiltration, with diffuse gadolinium retention in the myocardium and a faster gadolinium washout from the blood pool. This could make it difficult to determine the ideal myocardial inversion time (TI) in post-contrast pictures in order to get acceptable LGE images. For these situations, TI-scout sequences [54], early-to-late enhancement acquisitions [55], phase-sensitive inversion recovery (PSIR) LGE sequences [56], native T1 mapping [57], and extracellular volume fraction (ECV) [58] may be useful in making the diagnosis and identifying the stage of the disease.

The following sections will attempt to assess the various diagnostic approaches and their applicability in the detection of cardiac amyloidosis.

### 3.1. Electrocardiographic Evaluation in the Diagnostic Work-Up for CA

Even though the left ventricular wall width is thicker, the presence of a low-voltage electrocardiographic signal strongly suggests CA and can distinguish it from hypertensive or hypertrophic cardiomyopathy [59]. However, only 25% to 40% of ATTR-CA patients match the low-voltage criteria and may even meet the requirements for left ventricular hypertrophy [60]. Unknown factors may have caused the low-voltage ECG. Myocyte shrinkage and cardiac toxicity brought on by circulating light chains are potential contributing factors in AL amyloidosis [61]. In descriptive studies, more than half of the affected patients had an absence of R progression, sometimes known as a pseudo-infarction pattern, in the anterior precordial leads. However, only around 25% of patients had both a low ECG voltage and a pseudo-infarct pattern [62]. 

Artificial intelligence methods have been effective in deciphering electrocardiographic data to diagnose CA. A study obtained electrocardiographic maps of CA patients using ECG imaging. The complicated data sets collected from ECG data were then decoded using an ML approach, which also assisted in the creation of a straightforward surface ECG-based diagnostic algorithm for the identification of CA [63]. Two distinctive patterns were visible in the respective surface ECG leads. An ECG-based tool was created using thorough electroanatomic mapping of CA patients and an ML methodology. The ECG algorithm is straightforward and has been shown to be useful in detecting CA without the assistance of cutting-edge imaging modalities.

### 3.2. Echocardiography Evaluation in the Diagnostic Work-Up for CA

As reported above, CA is fatal, has a poor prognosis, and is frequently misdiagnosed as hypertrophic cardiomyopathy, which delays diagnosis [64]. The automated differentiation of two conditions using speckle tracking echocardiography was proposed. A recent study reported the findings of a great multicenter study assessing the diagnostic precision of a combination of traditional (non-deformation) and strain-derived echocardiographic variables in subjects with suspected CA. Authors derived two simple multiparametric scores to either identify or exclude CA in two different clinical settings: among subjects with established systemic AL amyloidosis or in subjects with a hypertrophic cardiac phenotype. Finally, they ascertained the functional and structural changes across the spectrum of gravity of amyloid deposition [65].

In order to assess the diagnostic benefit produced by AI techniques in the echocardiographic diagnosis of CA, patients with pathologically confirmed monoclonal immunoglobulin light chain cardiac amyloidosis and patients with hypertrophic cardiomyopathy were enrolled in a study. The most important indicators were found using ML models built using both conventional and cutting-edge technologies. The receiver operating characteristic curve (ROC) and area under the curve (AUC) were used to assess the performance. All models demonstrated excellent discriminative performance using clinical and echocardiography data. Support vector machine (AUC 0.95, *p* = 0.477), random forest (AUC 0.97, *p* = 0.31), and gradient boosting machine (AUC 0.98) showed comparable capacity to differentiate cardiac amyloidosis and hypertrophic cardiomyopathy when compared to logistic regression (AUC 0.91) [66]. The ability of data-driven machine learning to distinguish between two circumstances has been impressive. It can also automatically integrate a wealth of information to find the most discriminating predictors without making any assumptions. Automated ML can help identify patients with cardiac amyloidosis and enable prompt and efficient intervention, improving the prognosis in the big data era. 

A different study sought to create machine learning models with robust generalizability across numerous cohorts to detect HCM and distinguish it from other heart diseases using ECGs and echocardiograms [67]. On a data set with a realistic HCM prevalence, automated detection and manual interpretation were tested. The models distinguished between HCM and aortic stenosis, hypertension, and cardiac amyloidosis. Compared to cardiologists, automated HCM identification had a greater sensitivity in electrocardiography–echocardiography paired data analysis [67].

Finally, several other studies have attempted to automatically identify patients with unexplained left ventricular hypertrophy in electronic health record (EHR) data using computer methods such as text mining and ML [68,69,70].

### 3.3. Magnetic Resonance Evaluation in the Diagnostic Work-Up for CA

A common method for imaging the cardiovascular system for diagnosis and intervention is cardiac MRI (CMR). In addition to visualizing the anatomy, CMR has improved to the point that it can now be utilized to provide detailed quantitative measurements of the myocardium. These include physiologic measurements for mapping myocardial perfusion and blood volume, as well as relaxometry T1, T2, and T2* measures for the assessment of fibrosis, oedema, and iron as well as for assessment of tissue composition for the fat fraction [71]. A variety of disease states, such as diffuse interstitial or replacement fibrosis, oedema, or infiltrative diseases, can benefit from myocardial T1 mapping [72]. 

While in-patient regional or even temporal variations in native T1 may be informative of disease development or progression, cross-patient comparisons are less relevant due to the considerable overlap in myocardial native T1 levels between health and disease. The diagnostic value of T1 mapping for the quick identification of myocardial illness without the use of contrast agents would be improved by a method that could make use of the wealth of information included in myocardial T1 maps. The segmented anatomical volumes are converted into quantitative radiomic characteristics that, by examining the spatial relationship between (dis-)similar voxels (akin to terrain mapping), reveal details about tissue volume, shape, and texture [73,74,75,76,77,78,79].

The left ventricle is used as the main chamber for validating diagnoses. Recent research, however, has demonstrated that cardiac strain has discriminative value for all cardiac chambers and may help distinguish CA from HCM and healthy controls (CTRL) [80]. An experiment tested the hypothesis that strain measurements of the diagnostically less common cardiac chambers combined with general cardiac functional parameters may achieve a high CA diagnostic accuracy. This hypothesis was based on prior observations [81,82,83,84] and the desire to optimize CMR diagnostic accuracy for CA detection. The right ventricle and atria would therefore be the only sources of cardiac strain metrics.

By putting cardiac function and multi-chamber strain into supervised ML algorithms, the work challenges CA diagnoses [85]. CMR imaging was performed on CA patients, HCM participants, and healthy volunteers. Using the strain values for the left, right, and right ventricular atriums as well as heart function, the decision trees (DT), k-nearest neighbor (KNN), SVM linear, and SVM radial basis function (RBF) kernel algorithms developed a 41-feature matrix. Using linear SVM and RBF, a 10-feature principal component analysis (PCA) was carried out [85]. As a result, under supervised conditions, the SVM RBF kernel obtained competitive diagnostic accuracies. For non-contrast clinical decision-support systems in CA diagnostics, ML of multi-chamber cardiac strain and function may present unique perspectives.

However, other new demands are simultaneously placed on image analysis and reporting by CMR capabilities, opening up new prospects [86,87,88,89,90,91]. AI methods could speed up analysis times in addition to improving diagnostic accuracy. Higher patient throughputs, better objectivity, and reproducibility are all benefits of a fully automated solution. Cardiologists still primarily use manual delineation in clinical settings to evaluate heart function, viability, and tissue characteristics [92]. An expert’s thorough manual analysis can take between 9 and 19 minutes, according to recent research [93]. Convolutional neural networks (CNNs) in particular have been developed as DL models to automate CMR analysis and greatly shorten analysis times. CNN measurements have shown better reliability in multicenter studies [94], and they can be used to automatically interpret cardiac cine images to quantify the ejection fraction and other parameters with a performance level comparable to experienced readers. CNNs have been used to interpret and report on cardiac perfusion pictures obtained from MRI scanners [95]. In tests involving multiple centers, CNNs have also been constructed to measure LV function [96]. In a study, the LV blood volume and myocardium were segmented using a completely automated ML algorithm that was trained using 1923 images [97]. Performance of an ML algorithm was compared to that of three physicians (the “human”) and a commercial tool (cvi42, Circle Cardiovascular Imaging). Human analysis took longer (20 s per patient) than machine analysis (13 min). The combined dataset’s overall machine mis-segmentation rate was 1 in 479 pictures, which largely occurred in rare diseases that were not observed during training. Without correcting these segmentation errors, machine analysis outperformed three physicians in terms of precision, which resulted in a 46% decrease in the trial sample size needed to use an LVEF objective [97]. Consequently, a fully automated approach that outperforms human performance in terms of speed and accuracy can be used to measure LV structure and global systolic function.

The superiority of AI techniques was supported by additional investigations [98]. Using non-contrast cine CMR images, a study was conducted to see if texture analysis (TA) and ML-based classifications might be used in the differential diagnosis of CA [99]. Cine pictures were used to extract texture features. Nine ML models were created based on regression analysis with the least absolute shrinkage and selection operator (LASSO), and their diagnostic performance was assessed. The combined model including traditional MR metrics and radiomics texture characteristics performed better in terms of discrimination than the traditional MR metrics model. Additionally, the findings demonstrated that gray level non-uniformity (GLevNonU) levels in HCM patients were considerably greater than levels in CA patients and control groups. It has been proven that GLevNonU 25 can distinguish between CA and HCM patients. The discriminatory performance can be enhanced using the radiomics-MR combined model, and TA can be used to evaluate the changes in myocardial microstructure that take place across various phases of cardiomyopathies.

Another analysis produced similar outcomes. It was investigated in a study [100] whether radiomic characteristics from T1 maps obtained by CMR may improve the diagnostic utility of T1 mapping in separating health from disease and categorizing cardiac disease phenotypes. The type of heart illness was clearly associated with the first three major T1 radiomic components. The population’s unsupervised hierarchical grouping by myocardial T1 radiomics was substantially correlated with the type of cardiac illness [100].

In a subsequent investigation, CMR was carried out on participants who had suspected CA [101]. Three networks analyzed two-chamber, four-chamber, and short-axis LGE pictures (DL algorithms). When the average probability of CA from the three networks was 50% or less, the tags “amyloidosis present” or “absence” were assigned accordingly. The DL approach was contrasted with an ML algorithm taking into account all manually derived features to mimic an expert operator reading an exam. The DL approach demonstrated appropriate diagnostic precision. The diagnostic yield of an ML algorithm that took into account every CMR feature was comparable to a DL technique [101]. An ML-based strategy that simulates CMR reading by skilled operators and a DL approach evaluating LGE acquisitions both demonstrated comparable diagnostic performance for CA. However, there are a number of limitations to this study that must be acknowledged, including the fact that CA prevalence was very high. The study would also have benefited from an external validation cohort with a good representation of patients with hypertensive heart disease, hypertrophic cardiomyopathy, cardiac sarcoidosis, and other pathologies that could be mistaken for CA, as well as other limitations that are important to note. Additionally, because there were so few patients in the study, AL and ATTR cardiomyopathies were combined into a single diagnostic entity (CA).

Finally, three-dimensional myocardial deformation analysis, for example, has been incorporated into the computational analysis for the detection of the CA (3D-MDA). This parameter may provide suitable features for the training of ML-based models because it has been validated to provide uniform descriptors of cardiac architecture and deformation. Using automated disease identification to distinguish HCM from states that mirror it, such as CA, Anderson–Fabry disease, and hypertensive cardiomyopathy, was the goal of one study (HTNcm). To distinguish between HCM and other situations, a fully connected layer feed-forward neural network was developed [102]. This study revealed that it is possible to diagnose cardiomyopathy states using only 3D-MDA and that it is capable of differentiating between HCM and similar disease states. These results point to a promising future for computer-assisted diagnosis in clinical settings, and this finding that regional deformation factors add to global architectural and global functional aspects in determining illness etiology is in line with new data showing that these measures are closely related to underlying tissue characteristics [103]. In patients with HCM [104,105,106,107] and CA [108], as well as in non-hypertrophic conditions such as ischemic and dilated cardiomyopathy, strain-based markers have demonstrated a strong regional connection with underlying signs of fibrosis (i.e., interstitial expansion). As a result, there is potential for regionally encoded deformation indicators to offer novel insights into underlying myocardial tissue health and, in turn, provide pertinent data for the classification of illness genesis. 

### 3.4. Mass Spectrometry Evaluation in the Diagnostic Work-Up for CA

Immunohistochemical examination of biopsies from the affected organ or tissue has historically been used to determine the sub-type of the amyloidogenic protein [109,110]. However, because of its low sensitivity and low specificity [111,112,113], the latter of which is probably related to unspecific staining, this approach has been abandoned by many clinical pathology departments. However, utilizing immunohistochemistry (IHC), certain diagnostic facilities consistently attain a sensitivity higher than 90% [114]. Other recent techniques for classifying amyloidosis include mass-spectrometry-based shotgun proteome analyses of biopsies [115] and laser microdissection (LMD) of amyloid deposits visualized by Congo red staining combined with MS or immune electron microscopy (IEM) for the classification of localized amyloidosis [115,116,117]. In addition to measuring the amyloidogenic protein in question, mass-spectrometry-based approaches for amyloidosis subtyping also assess an amyloid protein signature that is common to all amyloidosis subtypes in distinct tissues in a very specific and quantitative manner.

A method for the objective detection of biopsies containing amyloid and amyloidosis subtyping was created by a study with the use of statistical models [118]. Clusterin, fibulin-1, vitronectin, complement component C9, three collagen proteins, as well as the well-known amyloid signature proteins apolipoprotein E, apolipoprotein A4, and serum amyloid P were discovered as novel “amyloid signature” proteins using a Boruta method applied on a random forest classifier to proteomics data obtained from the mass spectrometric analysis of Congo-red-positive amyloid-containing biopsies and Congo-red-negative biopsies in order to train an SVM learning system. When used on a blinded mass spectrometry validation data set of amyloid-containing biopsies that had been obtained, the trained algorithm outperformed controls in the distinction of amyloid-containing biopsies from controls, with an accuracy of 1.0. Additionally, in 102 out of 103 blinded cases, this technique successfully diagnosed amyloidosis patients according to the subtype [118]. With the objective and trustworthy classification of amyloid deposits and of the particular amyloid subtype, this model-assisted technique found novel amyloid-related proteins and proved the application of mass-spectrometry-based data in clinical diagnostics of disease (Table 2).

Finally, immunoelectron microscopy is a specific and extremely sensitive method for amyloid typing; it employs gold-labelled antibodies binding to amyloid fibrils. It can be utilized on formalin-fixed, paraffin-embedded material and is believed to be a new gold standard for amyloid typing next to mass spectrometry [119].

### 3.5. Medical Data and Radiomics in the Diagnostic Work-Up for CA

Beyond adopting a specific diagnostic method to identify CA, AI may be advantageous in using patient data for accurate and speedy diagnosis. Inpatient and outpatient hospital data, demographics, diagnoses, patient interactions and medical practices, prescriptions, laboratory tests, and charges are all included in the medical database, which is based in hospitals [120,121,122,123]. According to a study, using information from medical claims, a random forest machine learning model may spot prospective cases of wild-type transthyretin amyloid cardiomyopathy. In 1071 cases and 1071 non-amyloid heart failure controls, authors developed an ML model. Three nationally representative cohorts (9412 cases and 9412 matched controls) and a sizable single-center electronic health record-based cohort (261 cases and 39,393 controls) were used to validate the model [124]. The authors demonstrated that the ML model had good performance for detecting individuals with cCA in the derivation cohort and all four validation cohorts, giving a structured framework to raise suspicion of transthyretin CA in patients with heart failure.

An alternative study was conducted to create a routinely calculated set of laboratory-parameters-based expert-independent ML prediction model for CA [125]. The authors first created logistic-regression-based baseline linear models. In a subsequent phase, they performed non-linear prediction and enhanced their linear prediction model using an ML technique based on gradient tree boosting. The effectiveness of each diagnostic algorithm was then compared. A training cohort of patients with established CA and HF patients unrelated to amyloidosis served as the basis for all prediction models. The ROC AUC score for this model was 0.86, with sensitivity and specificity of 89.2% and 78.2%, respectively. This study reveals how ML enables the generation of a differentiated CA-related HF profile in comparison to HF patients without a CA connection using standard laboratory measures. This proof-of-concept study offers a potential new direction in the investigation of CA’s diagnosis and could support doctors’ clinical judgment.

## 4. Computational Methods for Amyloid Fibril Identification

The identification of the amyloid molecule is a critical step in the diagnostic procedure of a suspected amyloidosis. Immunohistochemistry and laser capture are the two most popular techniques for classifying amyloid fibrils, and mass spectrometry is then used to determine the amyloid subtype [114,115].

The amyloid fibrils appear to share a same structure regardless of the type of amyloid protein that makes them up. Therefore, it can be examined in vitro using X-ray diffraction techniques, electron microscopy, and certain chemical stains [126]. Due to the high volume of protein data generated by sequencing, computational approaches for detecting and forecasting fibrils have just recently evolved. However, these methods have cost and time constraints. Currently used protein sequencing methods run millions of reactions simultaneously while gathering enormous volumes of data.

The computational investigations have given us important atomic understanding. The “dock-and-lock” mechanism is a widely accepted explanation for fibril elongation that has been supported by extensive computational studies. In this mechanism, soluble monomers are quickly adsorbed on the fibril ends, primarily as a result of the entropy effect. This is followed by a slow structural rearrangement of the bound monomers, which is hampered by the need to break interactions between the monomers or misaligned monomer–fibril interactions. The process of the more recent discovery of fibril-dependent secondary nucleation is less well understood computationally. A proper interaction strength between the peptides and the fibril surface, the preference of the bound peptides for amyloidogenic conformations, the unrestricted movement of the peptides on the fibril surface, and their simple detachment for a quick turn-over are some of the key elements that simulation studies have so far identified as being crucial for an efficient secondary nucleation [127].

Doing more than just offering a molecular explanation for experimental findings regarding aggregation mechanisms is the ultimate goal of computational investigations.

Tian et al. [128] introduced the Prediction of the Amyloid Fibril-Forming Segment (Pafg) approach for finding fibril-forming segments in proteins based on a support vector machine (SVM). The Amino Acid Index database’s 41 physicochemical attributes were employed in their model (AAindex) [129]. With a Matthews correlation coefficient of 0.63, Pafg achieved an overall accuracy of 81%, a specificity of 80%, and a sensitivity of 82% [128].

Employing a different approach, RFAmyloid, a web server based on the SVMProt 188-D feature representation, the pse-in-one feature representation, and the random forest classifier, was presented as a method to discover amyloid proteins [130]. The Matthews correlation coefficient, sensitivity, specificity, and F-measure were 0.739, 0.781, 0.927, and 0.891, respectively, while the accuracy was 89.19%. 

Building an ML-based prediction model known as the PredAmylmultilayer perceptron led to similar outcomes. The authors integrated SVMProt-188D, tripeptide composition, and seven feature extraction techniques [131]. Other experiments used an accessible ReRF-Pred, a novel machine-learning approach based on a multi-feature encoding strategy to identify amyloidogenic regions utilizing the composition of tripeptides and pseudo amino acids [132,133,134]. A further attempt was performed using a linear SVM architecture on the Waltz database of 1415 total hexapeptides to classify amyloidogenicity patterns based on the short parts known as hot spots that might cause aggregation [135,136,137].

Because the datasets used in the literature are almost always unbalanced, a study was conducted to assess the classifier’s behavior solely on balanced amyloid fibril datasets. The automated learning of the various ML algorithms is influenced by the performance on balanced or unbalanced datasets [138]. More than 4000 chemical descriptors were used in the development of this new classifier, dubbed ENTAIL. With an accuracy on the test set of 81.80%, a sensibility of 100%, and a specificity of 63.63% on a balanced dataset, ENTAIL was based on the Naive Bayes Classifier with Unbounded Support and Gaussian Kernel Type. The investigation that was carried out showed that performance is superior in terms of performance on a balanced dataset [139] despite the various configurations of the tests.

In addition, researchers have predicted the solubility and located the aggregation hotspots within amyloid-forming proteins using sequence-based aggregation-scoring algorithms such as GAP, TANGO, WALTZ, PASTA, Aggrescan, FoldAmyloid, ANuPP, etc. [140,141,142,143,144,145,146,147,148]. These algorithms have made use of sequence- and structure-based properties, including patterns of hydrophobic and polar residues, charges, the capacity to form cross-motifs, scales for aggregation propensity derived from experimental data, solvent-exposed hydrophobic patches on molecular surfaces, and others. These algorithms’ benefits and drawbacks have been discussed elsewhere [149]. It has become widely accepted as a result of this research that the existence of an aggregation-prone region (APR) may be a prerequisite but not a necessary condition for protein aggregation. Other important parameters include the position of APRs in protein structure, the conformational stability of the native state, the conditions in the solution, and the kinetics of the aggregation process [150,151,152,153,154,155,156]. 

High individual antibody diversity is a significant problem for AL amyloidosis prediction and treatment. Although there are techniques for high-throughput antibody repertoire sequencing, it is not practical to experimentally determine each antibody’s amyloidogenicity. Therefore, it is essential to create computational techniques for quick and precise prediction of aggregating light chains. The scientific community needs to develop the computational algorithms now in use because they are not effective enough to estimate the solubility of antibodies and, in certain cases, only weakly correlate with conformational stability [157,158].

Common architecture and well-known binding locations in antibody light chains can offer crucial details for the prediction of amyloidogenicity under physiological settings. In a study, authors compared the hydrophobicity, presence of gatekeeper residues, disorderness, and other traditional sequence-based, aggregation-related features calculated for the CDR, FR, or VL regions of amyloidogenic and non-amyloidogenic antibody light chains. They then implemented the findings in a machine-learning-based webserver called “VLAmY-Pred” (https://web.iitm.ac.in/bioinfo2/vlamy, accessed on 21 February 2023). On a dataset of 1828 variable region sequences of the antibody light chains, the model exhibited a prediction accuracy of 79.7% (sensitivity of 78.7% and specificity of 79.9%) with a ROC value of 0.88 [159]. 

This model will be useful for a better understanding of the prognosis for patients who may likely have diseases brought on by light chain amyloidosis, for large-scale in silico analysis of antibody sequences produced by next generation sequencing, and, finally, for rational engineering of aggregation-resistant antibodies (Table 3).

## 5. Prognosis

Prognostic risk can also be determined by computational analysis carried out utilizing artificial intelligence tools. In order to identify patterns that correlate with amyloidosis type and prognosis, a study used an automated method to aggregate individual patients with cardiac amyloidosis as defined by 24 prespecified clinical characteristics [160]. The researchers gathered information from 1394 consecutive patients. The cohort included patients with AL, ATTRv, ATTRwt, and no definitive amyloidosis diagnosis. The 24 domains chosen for phenotyping the patients broadly defined their characteristics, including demographics (sex, age, and race), biometrics (body mass index and blood pressure), cardiovascular risk factors, New York Heart Association (NYHA) functional class, cardiac biomarkers, renal function, anemia, electrocardiographic features (low voltage and arrhythmia), echocardiographic variables (septal wall thickness, LV ejection fraction, and global longitudinal strain), and evidence of sarcoidosis. Diagnostic characteristics of ATTR amyloidosis and AL amyloidosis were also among the variables selected for characterization (scintigraphy uptake or cardiac fixation). Data were first transformed using principal component analysis to account for variations in qualitative and quantitative variables in order to produce continuous scores. Following this, the data were subjected to an unsupervised clustering analysis to produce self-organized maps from which a total of seven discrete clusters were defined. After being placed on these clusters, patients with known amyloid types had their prognosis (4-year mortality) reported. Low global longitudinal strain (mean 8.4%) and LV ejection percent (mean 41%), low voltage, and monoclonal gammopathy were the characteristics of Cluster 1, which also had the highest levels of cardiac biomarkers and the highest NYHA functional class. Unsurprisingly, Cluster 1 had AL amyloidosis in 88%, accounting for 67% of all patients with AL amyloidosis. Older males, soft tissue deposits, the thickest LV septum, and the highest rates of bone-seeking radiotracer uptake were all present in Clusters 2 to 4. Clusters 2, 3, and 4 received the majority of the mapping for ATTRwt patients. These clusters differed from one another, allowing for additional differentiation. While Cluster 3 patients had arrhythmia but fewer cardiovascular risk factors, Cluster 4 patients had a high proportion of risk factors but preserved biochemical and functional parameters, and Cluster 2 patients had a higher proportion of cardiovascular risk factors and impaired biochemical and functional parameters. When survival was taken into account, these variations became significant. Comparing Clusters 3 and 4 (about 55% survival) to Cluster 2, ATTRwt patients had a 20% survival rate. In actuality, the survival rate for ATTRwt patients in Cluster 2 was lower (42% survival) than that of AL patients in Cluster 1. Patients with ATTRv amyloidosis were more uniformly spread out among Clusters 2 to 4 as well as Cluster 6, which reflects the variation in phenotypic manifestation (cardiomyopathy and neuropathy) between the various TTR variants and the ages of ATTRv patients. Patients with nonamyloid disease were discovered in Clusters 5 to 7, with Cluster 7 standing out because patients there had greater body mass indices, diabetes, and dyslipidemia than in the other clusters, which is consistent with the profile of metabolic heart disease. In Cluster 7, the four-year death rate was 31% (69% survival) [160]. It is important to note the study’s shortcomings. First, the investigators admitted that a sizeable amount of the missing data (up to 33% for scintigraphy) was handled via imputation, which is formally correct but could eventually draw spurious results. Second, using the same dataset, the clustering analysis was created and then examined for relationships with amyloid type. Third, not all of the analyses were unsupervised. Fourth, no information about the medical care obtained is provided. Without understanding the medicines used and their results, it is challenging to assess survival data [161]. 

## 6. Conclusions

Although there has been significant progress in the diagnosis and management of cardiac amyloidosis, there are still significant knowledge and diagnostic process gaps. Nuclear scintigraphy utilizing technetium-labelled bone-seeking radiotracers for ATTR cardiac amyloidosis has become widely available, which has facilitated diagnoses, although improper patient selection and interpretation continue to happen, leading to erroneous and/or missing diagnoses [162,163]. Furthermore, there is significant phenotypic overlap in the echocardiographic, cardiac magnetic resonance, and biomarker profiles of the most prevalent cardiac amyloidosis types of AL, ATTRwt, and variant transthyretin [ATTRv], which makes proper adjudication difficult. 

A conclusive diagnosis of HCM is made using an echocardiography as well as cardiac MRI [164]. Due to the underdiagnosis of HCM, typical workflows involving these diagnostic modalities are obviously insufficient. This is due to factors such as cost, the subtlety of early disease findings, the invasiveness of the approach, the availability of the modality, as well as the requirement that non-specialist providers either remember the necessary diagnostic steps or make a timely referral. HCM can also be identified by distinctive ECG alterations [165,166,167,168,169], a method that is more generally accessible. 

Several studies have developed ML strategies to detect HCM from both electrocardiography and echocardiography [170,171,172,173], but to date, these approaches have only involved a single modality and have only been evaluated at single centers.

Unfortunately, CA lacks a large amount of data when compared to other cardiovascular conditions, making the development of reliable AI-based models difficult and somewhat poorly scalable and generalizable. However, new frontiers of AI are advancing in this sense, with the development of novel principles and the practical application of well-grounded methods that are yet neglected or poorly used in medicine, which can promote a full adoption of AI in CA. Such methods, which are of different natures, include, for example, transfer learning, commonly used in scientific and technological realms, or federated learning, which enables huge amounts of data to be collected from various sources and also features overall superior safety and security for patients and their sensitive data.

Increased generalizability requires the use of vast amounts of data from various institutions. However, training data in the medical area could include personally identifiable information, making sharing difficult. An ML technique known as federated learning enables the training of an ML model using data sets from other institutions without sharing the raw data. Such an approach was never explored directly in CA, but the success demonstrated in HCM detection and differential diagnosis with respect to cardiac disorders featuring somewhat similar clinical characteristics, including CA, suggests its likely application in future studies dealing with CA [67]. This would benefit researchers and clinicians from several points of view, including greater data availability and the possibility to guarantee the patients whose data are collected with higher degrees of data protection and privacy regulations.

Accurate diagnosis is a prerequisite for individualized medicine, which necessitates analysis that goes beyond the data encoded in the systems utilized in clinical practice [174]. Clinical researchers have taken a keen interest in this area with the surge of electronic health records (EHRs), as the comprehensive and extensive use of clinical datasets holds enormous promise for revolutionizing the healthcare system. For instance, nursing records give information on patient conditions, and the data frequently reflects nurses’ cumulative knowledge of how to evaluate physiological and psychological signs to identify patient deterioration.

The learning and training approaches of the algorithm are based on the inpatient clinical (medical and nursing) records employed for a study. The strategy involved treating the data produced by each admission and discharge occurrence as data vectors in order to facilitate data aggregation. The high dimensionality of the data was inferred by the vast number of clinical histories, and the absence of a diagnosis resulted in a significant class imbalance because the condition was not common. Despite the small number of patients with amyloidosis in this study, the suggested method shows that it is still possible to learn from clinical records in the absence of data. The algorithm originally used data from the overall research population during the validation phase. Then, it was used on a sample of people with heart failure. The findings showed that when data vectors profile each disease occurrence, the algorithm may detect disease. The prediction levels demonstrated that this method may be helpful in illness screening procedures on a particular group [175].

Given the trends noticed in medicine concerning the use of AI, and in particular in the cardiovascular field and in the CA diagnosis and detection, it is hypothesized that the number of scientific products related to this subject is probably destined to grow in the next few years. However, aside to the important scaling up of AI-related research, which is particularly useful to lead to new discoveries and important novelties and insights about physiopathological and etiopathological signs of the disease, important gaps are still present in the translation from bench to bedside of AI and technological advancements in general in every sector of medicine [176]. However, with the advent of new AI-based tools, which are pervasively entering everyday life (e.g., ChatGPT or similar programs), it is predictable that such novel approaches will be more often scaled into the clinical practice in the next few years, which will have significant benefits for the clinical community.

In conclusion, for the two most prevalent kinds of cardiac amyloidoses (ATTR and amyloidogenic light chain), disease-modifying therapies are now available, and earlier treatment in the course of the disease may be associated with a higher treatment response [177]. Therefore, it is crucial to diagnose cardiac amyloidosis early and more thoroughly. In any event, it is crucial to diagnose CA as soon as possible since, if left untreated, the median survival time after the onset of HF is just 6 months [178], even if contemporary treatments can prolong life by several years and put the disease into a protracted remission [179]. The prognosis of patients with AL-CA has significantly improved as a result of the availability of proteosome-inhibiting drugs, particularly bortezomib, which is frequently coupled with dexamethasone and low-dose cyclophosphamide. These factors make early identification of the illness a crucial clinical requirement. In the past few years, early diagnoses of the disease have resulted from the active screening for CA using new AI techniques [180]. This approach could guarantee an overall more adequate management of the pathology.

## Figures and Tables

**Table 1:** Some forms of cardiac amyloidosis.

Amyloid Type	Protein	Distribution	Etiology	Diseases Associations
AL	Immunoglobulin light chain	Systemic and localized	Acquired	Plasma cell dyscrasia
AH	Immunoglobulin heavy chain	Systemic	Acquired	Plasma cell dyscrasia
ATTR	Transthyretin	Systemic	Acquired and hereditary	
ATTRwt	Wild-type transthyretin	Systemic	Acquired	Aging
ATTRv	*TTR* gene variant	Systemic	Hereditary	
AA	Serum amyloid A	Systemic	Acquired	Chronic inflammation
Aß2M	ß2-microglobulin	Systemic	Acquired and hereditary	Chronic hemodialysis
AANF	ANF	Localized	Acquired	Atrial fibrillation

**Table 2:** Possible ML approaches in the diagnosis of cardiac amyloidosis.

Technique	Parameters	Population	IA Method	Ref.
Electrocardiographic evaluation	R-peak time	Treatment-naïve patients with CA	Unsupervised machine learning approach	[63]
Echocardiography data	Speckle tracking echocardiography	Monoclonal immunoglobulin light chain cardiac amyloidosis and patients with hypertrophic cardiomyopathy	Support vector machine, random forest, and gradient boosting machine	[66]
	Time series of voltages recorded for 10 seconds at 250 Hz Echo videos standardized to 30 frames with 30 frames per second	Patients with hypertrophic cardiomyopathy	A federated learning approach	[67]
Magnetic resonance	Multi-chamber strain and heart function	Patients with hypertrophic cardiomyopathy	Decision tree, k-nearest neighbor, SVM linear, and SVM radial basis function kernel algorithm processing	[85]
	Left ventricular cavity and myocardium right ventricle	Adenosine stress and rest perfusion scans	A convolutional neural network	[95]
	Cine MRI images of left ventricle	Data from three major MR vendors	Convolutional neural networks	[96]
	Left ventricle blood volume and myocardium	Images from 1932 patients with multiple diseases from multiple centers	Convolutional neural networks	[97]
	Left ventricular volume in the end-systolic images	Patients with CA, HCM, and normal subjects	K-nearest neighbor, random forest, naïve Bayes, support vector machine, logistic regression, and artificial neural networks	[99]
	T1 mapping	Normal subjects, patients with left ventricular hypertrophy of various causes, patients with HCM, and patients with known cardiac amyloidosis	A random forest machine learning algorithm	[100]
	Biventricular systolic function	Subjects with suspected CA, unexplained left ventricular hypertrophy with blood dyscrasia and suspected light-chain amyloidosis	Three base convolutional neural networks	[101]
	Volumetric and strain markers	Patients from the Cardiovascular Imaging Registry of Canada	A neural-network-based model	[102]
Mass spectrometry	Clusterin1, fibulin-1, vitronectin, complement, apolipoprotein E	Congo-red-positive and -negative amyloid-containing biopsies	A Boruta method applied on a random forest classifier	[118]

**Table 3:** Computational methods for amyloid fibril identification.

Method	Target	IA	Results	Ref.
Pafig	Hexpeptides associated with amyloid fibrillar aggregates	Support vector machine	Accuracy of 81% and Matthews correlation coefficient of 0.63	[128]
Pse-in-One	DNA, RNA, and protein sequences	Support vector machine and neural network	Matthews correlation coefficient 0.739, sensitivity 0.781, specificity 0.927, F-measure 0.891, and accuracy 89.19%	[130]
PredAmylmultilayer	Protein sequences	Waikato environment for knowledge analysis	Accuracy 91.59%, specificity 0.950, and sensitivity 0.836	[131]
ReRF-Pred	Composition of tripeptides and pseudo amino acids	Amyloidogenic regions	Accuracy 0.828, specificity 0.921, and Matthew correlation coefficient 0.619	[132]
ENTAIL	Protein precursors	Machine learning	Accuracy 81.80%, sensibility 100%, and specificity 63.63%	[139]
VLAmY-Pred	Tripeptides composition	Random forest	Accuracy 79.7%, sensitivity 78.7%, and specificity 79.9%	[159]

## Data Availability

Not applicable.
